# The Validity and Reliability of the Sinhala Translation of the Patient Health Questionnaire (PHQ-9) and PHQ-2 Screener

**DOI:** 10.1155/2014/768978

**Published:** 2014-03-27

**Authors:** Raveen Hanwella, Shakya Ekanayake, Varuni A. de Silva

**Affiliations:** Department of Psychological Medicine, Faculty of Medicine, University of Colombo, Kynsey Road, 08 Colombo, Sri Lanka

## Abstract

The Patient Health Questionnaire (PHQ-9) was adapted and translated into Sinhala. Sample consisted of 75 participants diagnosed with MDD according to DSM-IV criteria and 75 gender matched controls. Concurrent validity was assessed by correlating total score of PHQ-9 with that of Centre for Epidemiological Studies Depression Scale (CESD). The Structured Clinical Interview for DSM-IV (SCID-II) conducted by a psychiatrist was the gold standard. Mean age of the sample was 33.0 years. There were 91 females (60.7%). There was significant difference in the mean PHQ-9 scores between cases (14.71) and controls (2.55) (*P* < 0.001). The specificity of the categorical algorithm was 0.97; the sensitivity was 0.58. Receiver operating characteristic (ROC) analysis found that cut-off score of ≥10 had sensitivity of 0.75 and specificity of 0.97. The area under the curve (AOC) was 0.93. The sensitivity of the two-item screener (PHQ-2) was 0.80 and the specificity was 0.97. Cronbach's alpha was 0.90. The PHQ-9 is a valid and reliable instrument for diagnosing MDD in a non-Western population. The threshold algorithm is recommended for screening rather than the categorical algorithm. The PHQ-2 screener has good sensitivity and specificity and is recommended as a quick screening instrument.

## 1. Introduction

The Global Burden of Disease Study 2010 states that mental and behavioural disorders are a main contributor to Years Living with Disability (YLD) [1]. Patients with depressive symptoms present to primary care settings, specialized care units, and psychiatry treatment services [2, 3]. A WHO multicountry study reported that the prevalence of depression in primary care was 14% [4]. Depression is underdiagnosed in primary care and specialized treatment settings and only about half the patients with depression are accurately diagnosed by general practitioners [5, 6].

The Patient Health Questionnaire (PHQ-9) was developed as a screener for depression during the development of PRIME-MD [7, 8]. It is a self-administered tool based on DSM-IV criteria for diagnosing depressive disorder. It can be used to monitor severity of depression by scoring the frequency of each symptom on a scale of 0–3. It can also be used to diagnose major depressive disorder (MDD).

The PHQ-9 has been used in a variety of settings. It has been translated and culturally adapted for diagnosing depressive disorder in many countries [9–11]. A meta-analysis reported that the summary sensitivity of the PHQ-9 was 0.77 (0.71–0.84) and specificity was 0.94 (0.90–0.97) [12]. The PHQ-2 is used as a screening tool for depression in primary care, and patients who screen positive are subject to further evaluation [8, 13].

This study had two main aims. The first was to establish the validity and reliability of the PHQ-9 in a Sri Lankan population. Sri Lanka has a shortage of psychiatrists and many patients with depression are treated in nonpsychiatric settings [14]. Therefore a valid and reliable depression screening instrument is invaluable in these settings. The second aim was to compare the sensitivity and specificity of the different diagnostic algorithms of the PHQ-9 which would help identify the best algorithm for diagnosis of MDD.

## 2. Materials and Methods

### 2.1. Sample

Sample size was calculated assuming a sensitivity and specificity of 0.85. Sample consisted of 75 cases diagnosed with major depressive disorder and 75 gender matched controls. Cases were selected from an outpatient psychiatry clinic in a tertiary care hospital in Colombo, Sri Lanka. Patients are referred to this clinic from other units in the hospital. Patients also directly seek treatment from this clinic. Therefore the patient population is comparable with a primary care population. Controls were selected from the community following a screening assessment to exclude depressive disorder. Patients with bipolar depression were excluded from the study.

### 2.2. Study Procedure

The study methodology has been described in a previous publication [15]. A combined qualitative and quantitative approach was used for the translation of the PHQ-9 [16]. A panel of six experts who were bilingual individually translated the scale into Sinhala. Sinhala is a language spoken by about 75% of Sri Lankans. The translations were then discussed in a group consisting of all six experts. The best translation for each item of the scale was decided by consensus of the group. The final translated scale was back translated to English by a bilingual expert who was unaware of the original scale. The back translated scale was compared with the original scale. The translated scale was pretested on a group of 20 people in the community.

Major depressive disorder was diagnosed based on the Structured Clinical Interview for DSM-IV Disorders (SCID-1) [17]. Cases and controls completed the Sinhala version of the PHQ-9 questionnaire and the Centre for Epidemiological Studies Depression Scale (CESD) [15]. The CESD was used to assess the concurrent validity.

Written informed consent was obtained from all participants and ethical approval was obtained from the Ethics Review Committee of the Faculty of Medicine, University of Colombo.

### 2.3. Measures

The Patient Health Questionnaire is a nine-item instrument that assesses symptoms of depression as listed in the DSM-IV. Each of the nine items is scored from 0 (not at all) to 3 (nearly every day). The total scores can range from 0 (no depressive symptoms) to 27 (all symptoms occurring daily). The PHQ-9 uses two diagnostic algorithms to diagnose MDD. The categorical algorithm requires “more than half the days” or “nearly every day” response to at least five questions which should include question 1a or 1b or both. Question 1i is counted as positive if the thought is present on several days [18]. The second algorithm uses a threshold score for diagnosis. The total score also indicates the severity of depression; scores of 0 to 4 represent a minimal level of depression; 5 to 9, mild; 10 to 14, moderate; 15 to 19, moderately severe; and 20 to 27, severe. In addition the first two questions of the PHQ-9 can be used as a screener for depressive disorder (PHQ-2) [13].

### 2.4. Statistical Analysis

Statistical analysis was done using SPSS Statistics version 18.0 [19]. Internal consistency was measured using Cronbach's alpha. Criterion validity was assessed using receiver operating characteristic (ROC) analysis which gave the sensitivity and specificity of the PHQ-9 at different cut-off points. The Structured Clinical Interview for DSM-IV (SCID-I) conducted by a psychiatrist was used as the gold standard [17]. Concurrent validity was assessed by correlating the total scores of CESD and PHQ-9. The sensitivity and specificity of the two algorithms of the PHQ-9 and the two-question screener (PHQ-2) in diagnosing MDD were assessed.

## 3. Results

The sample consisted of 75 cases and 75 controls. The mean age of the sample was 33.0 years. There were 91 females (60.7%). The controls (28.33 years) were significantly younger than the cases (37.51 years) (*t* = 3.48, *df* = 118, and *P* = 0.001). There was no significant difference in gender distribution between cases and controls (**χ** ^*2*^ = 1.45, *df* = 2, and *P* = 0.485).

The mean PHQ-9 total score of the sample was 8.67 (SD 8.22). There was significant difference in the mean PHQ-9 scores between cases (14.71) and controls (2.55) (*t* = 13.58, *df* = 149, and *P* < 0.001). Classification of cases according to the severity of depression based on the PHQ-9 total score showed that 7 (9.2%) had minimal depression (score 1–4), 12 (15.8%) mild depression (score 5–9), 15 (19.7%) moderate depression (score 10–14), 20 (26.3%) moderately severe depression (score 15–19), and 22 (28.9%) severe depression (score 20–27). Of the controls 61 (81.3%) had minimal depression, 12 (16%) had mild depression, one had moderate depression and another had moderate to severe depression, and none had severe depression.

### 3.1. Validity

The Structured Clinical Interview for DSM-IV Disorders (SCID-1) was used as the “gold standard” [17]. When the categorical algorithm was used to diagnose major depressive disorder, the sensitivity was 0.58 and the specificity was 0.97 (Table 1).

**Figure 1:**
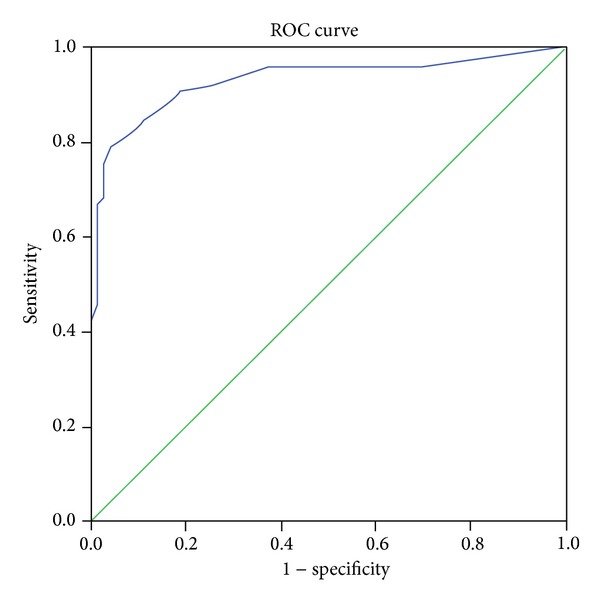
Receiver operating curve. Diagonal segments are produced by ties.

Receiver operating characteristic (ROC) analysis identified sensitivity and specificity at different cut-off points for the diagnostic algorithm using the total score (Figure 1). The area under the curve (AOC) was 0.93. Cut-off score of ≥10 gave a sensitivity of 0.75 and specificity of 0.97 (Table 2).

Concurrent validity was assessed by correlating the total scores of PHQ-9 and Centre for Epidemiological Studies Depression Scale (CESD). The Pearson correlation coefficient was 0.87.

In the two-item categorical algorithm, depression screening is positive if one or more of the two depressive symptom criteria are present. The sensitivity of the two-item screener was 0.80 and the specificity was 0.97 (Table 3).

### 3.2. Reliability

Cronbach's alpha was 0.90. The mean item scores and corrected item-total correlations are given in Table 4. The mean scores of the items ranged from 0.57 to 1.36. The lowest item mean (0.57) and the lowest item-total correlation (0.44) were for item 6* Feeling bad about yourself or that you are a failure*. Cronbach's alpha, if item is removed, reduced for all items.

## 4. Discussion

This study examined the validity and reliability of two algorithms of the PHQ-9 and the two-question screener (PHQ-2) in diagnosing major depressive disorder. When the categorical algorithm was used, the sensitivity was 0.58 and the specificity was 0.97. When the threshold algorithm was used, a cut-off score of ≥10 gave a sensitivity of 0.75 and specificity of 0.97. Cronbach's alpha was 0.90 which may indicate the unidimensionality of the scale. The sensitivity of the two-item screener (PHQ-2) was 0.80 and the specificity was 0.97.

When the categorical algorithm was used, the PHQ-9 had very high specificity but low sensitivity. There are reports that the categorical algorithm results in low sensitivity (0.42–0.53) but high specificity [11, 20, 21]. The sensitivity and the specificity of a diagnostic test depend on the characteristics of the test and the population in which it is used [22]. Sensitivity is higher when the sample consists of more patients with severe disease. In our sample, although the mean PHQ-9 score was (8.67) higher than that in several other studies, this did not result in high sensitivity.

It is possible that in some cultures emotional problems are expressed differently and this can influence the interpretation of scale items. However low sensitivity was seen with the categorical algorithm but not the threshold algorithm. Therefore the low sensitivity of the categorical algorithm may reflect the stringency of criteria for diagnosis rather than problems with interpretation of items. Similar findings have led other researchers to recommend the use of the threshold algorithm rather than the categorical algorithm [11, 20].

It is thought that patients from non-Western cultures are less likely to acknowledge the presence of low mood. Patients with depressive disorder, from both Western and non-Western cultures, have been found to present initially with somatic symptoms such as musculoskeletal pain and fatigue [23]. The mean score of items of the PHQ-9 in our sample showed that somatic symptoms of poor sleep and lack of energy were commonly acknowledged, but the item most frequently reported by the sample was low mood. Therefore, in our sample, regardless of the presenting complaint, patients with depressive disorder did acknowledge experiencing low mood. This finding has been reported from a study in Thailand too [11].

The PHQ-2 screener had high sensitivity and specificity. The sensitivity of the two-item screener (0.80) was higher than that of the categorical algorithm (0.58) and the threshold algorithm (0.75). The specificity was the same as the other two algorithms.

The United States Preventive Services Taskforce recommends using the first 2 questions in the PHQ-9 “Over the past 2 weeks, have you felt down, depressed, or hopeless?” and “Over the past 2 weeks, have you felt little interest or pleasure in doing things?” in screening for depression in adults because it may be as effective as using more formal instruments [24, 25]. Our findings show that the PHQ-2 is effective in screening for depression as it has good sensitivity and specificity and can be administered in busy outpatient settings with ease. However it is not recommended for diagnosis of major depressive disorder.

Our study has several limitations. We used a case-control design which is known to increase the sensitivity and specificity of the instrument [22]. However the patient sample included an appropriate spectrum of mild and severe disease as well as treated and untreated individuals. A major limitation of the study was that we recruited patients from a tertiary care psychiatry unit. Although this outpatient clinic treated patients who directly present similar to a primary care facility the composition of the patient population would be different to that of a primary care centre.

Patients presenting to primary care services may be diagnosed with specific clinical syndromes that vary in duration and severity over time and also encompass an admixture of somatic and psychological symptoms that do not match current psychiatric diagnostic systems [26]. This is especially true for depressive symptoms. For example, pain may be a presenting symptom of depressive disorder in primary care. Therefore instruments and diagnostic criteria may need to be adapted for use in primary care.

## 5. Conclusions

We recommend the use of the threshold algorithm rather than the categorical algorithm for screening for depressive disorder, because of the better sensitivity of the former. We also recommend the use of the PHQ-2 screener in all clinical settings, because it has high sensitivity and specificity and can be administered easily.

## Figures and Tables

**Table 1:** Sensitivity and specificity of the PHQ-9 categorical algorithm.

	Cases	Controls
PHQ-9 positive	44	2
PHQ-9 negative	32	73

**Table 2:** Sensitivity and specificity of PHQ-9 at different cut-off scores.

Cut-off score	Sensitivity	Specificity
≥5	0.91	0.81
≥6	0.88	0.84
≥7	0.84	0.89
≥8	0.82	0.92
≥9	0.79	0.96
≥10	0.75	0.97
≥11	0.68	0.97
≥12	0.67	0.99
≥13	0.58	0.99
≥14	0.57	0.99
≥15	0.55	0.99
≥16	0.50	0.99

**Table 3:** Sensitivity and specificity of the PHQ-2 screener.

	Cases	Controls
PHQ-2 positive	61	2
PHQ-2 negative	15	73

**Table 4:** PHQ-9 item mean and item-rest correlation.

	Mean	Standard deviation	Corrected item-total correlation	Cronbach's alpha if item is removed
Item 1 Little interest or pleasure in doing things	0.92	1.28	0.73	0.88
Item 2 Feeling down, depressed, or hopeless	1.36	1.30	0.74	0.88
Item 3 Trouble falling or staying asleep or sleeping too much	1.25	1.32	0.71	0.88
Item 4 Feeling tired or having little energy	1.30	1.32	0.76	0.88
Item 5 Poor appetite or overeating	0.97	1.25	0.60	0.89
Item 6 Feeling bad about yourself or that you are a failure	0.57	1.01	0.44	0.90
Item 7 Trouble concentrating on things	0.82	1.26	0.62	0.89
Item 8 Moving or speaking so slowly that other people could have noticed	0.83	1.27	0.70	0.88
Item 9 Thoughts that you would be better off dead or of hurting yourself	0.64	1.05	0.64	0.89
